# Clinical Characteristics and Follow-Up of 19 Children With Hashimoto’s Thyroiditis Aged Below 3 Years: A Single-Center Retrospective Analysis

**DOI:** 10.3389/fendo.2021.737527

**Published:** 2021-09-03

**Authors:** Shi Tang, Min Yang, Dan Zhang, Ya-jie Tong, Ying Xin

**Affiliations:** Department of Pediatrics, Shengjing Hospital of China Medical University, Shenyang, China

**Keywords:** Hashimoto’s thyroiditis, children, hypothyroidism, levothyroxine, global developmental delay

## Abstract

**Aim:**

To analyze the clinical characteristics of Hashimoto’s thyroiditis (HT) in children below 3 years of age in order to improve the understanding of the disease, avoid misdiagnosis, and achieve early diagnosis and treatment.

**Methods:**

The study retrospectively analyzed the clinical data of 19 patients diagnosed with HT in the first three years of life.

**Results:**

The patients (12 female, 7 male) had an average age of 26.1 ± 8.2 months (range 10–36 months). At presentation, one patient had euthyroidism, ten had hypothyroidism, seven had subclinical hypothyroidism, and one had hyperthyroidism. The most common reasons for doctor’s visits were thyroid enlargement (21.1%), global developmental delay (21.1%), and routine thyroid function tests in patients with type 1 diabetes (26.3%). Sixteen patients provided follow-up data, and the mean follow-up time was 23.31 ± 16.44 months (range 1–48 months). In the hypothyroidism group, one patient stopped levothyroxine (LT4) treatment after 2 months; the remaining patients had been treated with LT4 since their diagnosis. In the subclinical hypothyroidism group, one patient whose thyroid function returned to normal after 1 month of being diagnosed was not treated. The remaining patients received LT4 treatment at their diagnosis or during follow-up. The patient with hyperthyroidism was treated with methimazole after diagnosis, but treatment was discontinued 11 months later and LT4 was initiated 26 months after diagnosis. One in four patients with global developmental delay approached normal mental development after LT4 treatment. Four in six patients with short stature achieved height catch-up.

**Conclusion:**

At their initial HT diagnosis, most of the children showed hypothyroidism or subclinical hypothyroidism. Children with global developmental delay require continual screening, even if the thyroid function is normal after birth, to determine whether they have HT-induced hypothyroidism. Thyroxine replacement could partially relieve the clinical manifestations of hypothyroidism and early diagnosis and treatment are essential for improving patient prognosis.

## Highlights

➢Most children below 3 years with Hashimoto’s thyroiditis are in the hypothyroid or subclinical hypothyroid stage and need timely treatment.
➢Boys have a higher risk of Hashimoto’s thyroiditis within the first 2 years of life
➢Global developmental delay is the characteristic and serious clinical manifestation of children under 3 years old with Hashimoto’s thyroiditis.
➢Short stature and poor appetite may be overlooked by parents of young children with Hashimoto’s thyroiditis, leading to a delay in diagnosis and treatment.
➢Thyroxine replacement could partially relieve the clinical manifestations of hypothyroidism.

## Introduction

Hashimoto’s thyroiditis (HT), also known as chronic lymphocytic thyroiditis, is the most common thyroiditis that occurs in children and adolescents and is predominantly diagnosed in females. The disease incidence gradually increases with age and reaches its peak during adolescence (1, 2).

HT is an organ-specific autoimmune disease. In individuals with a specific genetic predisposition, environmental factors such as stress, infection, iodine intake, and vitamin-D deficiency trigger an immune response through thyroid antigens, facilitating the onset of HT (3, 4). HT can be diagnosed by the presence of anti-thyroid antibodies against peroxidase (TPOAb) and/or anti-thyroid antibodies against thyroglobulin (TGAb) and is confirmed by typical alterations observed in a thyroid ultrasound (5).

The main reason for patients with HT to see a doctor is goiter (i.e., abnormal enlargement of the thyroid) (6). However, the clinical manifestations are very heterogenous, and abnormal thyroid function or elevated thyroid autoantibody levels are often recognized when addressing other clinical symptoms (6). At the time of diagnosis, the patient’s thyroid function may vary, mainly in cases of euthyroidism, hypothyroidism, and subclinical hypothyroidism (7), and occasionally in cases of subclinical hyperthyroidism (6, 8).

HT is very rare in children under 3 years old, and previous reports are mostly case studies (9–18); therefore, the clinical data available on the disease course in such children are limited. This study summarizes the clinical data of 19 patients with HT under the age of 3 years, with the aim of facilitating the analyses of the clinical characteristics of HT in children under 3 years old, improving disease awareness, avoiding misdiagnosis, promoting early identification and treatment, and improving overall patient prognosis.

## Materials and Methods

### Subjects

This study reviewed the clinical data of all patients who were diagnosed with HT before the age of 3 years, at the Pediatric Endocrinology Department of Shengjing Hospital Affiliated with China Medical University, between September 2011 and February 2021. A total of 19 patients were included. The inclusion criteria were as follows: (1) age < 3 years; (2) TGAb and/or TPOAb levels exceeding 40 IU/mL and 35 IU/mL, respectively; and (3) thyroid ultrasound results being aligned with changes seen in autoimmune thyroid disease; (4) negative result of screening for congenital hypothyroidism. Growth failure was considered as short stature and/or abnormally low height velocity (19). Short stature was defined as height was under 2SD of average height in same race, gender and age (20). Global developmental delay was diagnosed if the patient significant delayed in two or more developmental domains of gross or fine motor, language, cognition, social and activities of daily living (21).

The patients were classified into the following groups according to their thyroid function at the time of diagnosis: (1) overt hypothyroidism [low free thyroxine (FT4) and elevated thyroid-stimulating hormone (TSH)]; (2) subclinical hypothyroidism (normal FT4 and elevated TSH); (3) euthyroidism (FT4 and TSH within normal limits); and (4) hyperthyroidism (high FT4 and low TSH). Of the 19 patients, 16 were followed-up, with the average follow-up period being 23.31 ± 16.44 months (range 1–48 months).

### Methods

The information of each patient, including age, sex, chief complaint, clinical manifestations, family history, other findings, treatment, follow-up, recumbent length, weight, and developmental quotients (DQ) was collated. The data of biochemical parameters including free triiodothyronine (FT3), FT4, TSH, TPOAb, TGAb, red blood cell, hemoglobin, aspartate aminotransferase (AST), alanine transferase (ALT), bilirubin, creatine kinase (CK), creatine kinase MB isoenzyme (CK-MB), urea, creatinine, cholesterol, and triglycerides were recorded. The result of thyroid ultrasound and cardiac ultrasound were collected as well.

Recumbent length and weight were measured with the patients wearing light clothes and not wearing shoes; recumbent length was measured in the lying position. The age and gender z-scores of recumbent length and weight refer to standard growth values and standardized growth curves of the recumbent length and weight of Chinese children under the age of 7 years in 2009 (22). Tsumori-Inage Developmental Test was administered to estimate patients’ DQ.

A chemiluminescent immunoassay was used to detect changes in the expression of FT3, FT4, TSH, TPOAb, and TGAb. Being positive for thyroid autoantibodies was defined as having a TGAb level > 40 IU/mL and/or a TPOAb level > 35 IU/mL (23).

Thyroid ultrasound was used to observe thyroid echo, size, and the presence of thyroid nodules. The volume of the thyroid was calculated using the formula proposed by Delange et al. (24): volume (mL) = 0.479 x L (cm) x D (cm) x W (cm) (L refers to the length of the thyroid, D refers to the depth, and W refers to the width). Cardiac ultrasound was mainly used to observe structure and function and to evaluate the presence of pericardial effusion.

### Statistical Analysis

Data were expressed as mean ± standard deviation (X ± SD) or absolute and relative frequencies. Descriptive statistics was performed using the SPSS 21.0 statistics software (IBM, Chicago, IL, USA).

## Results

### General Condition

The patient cohort included 12 female and 7 male patients (female-to-male ratio of 1.7:1) with a mean age of 26.1 ± 8.2 months (range 10–36 months). 1648 pediatric patients (0-14 years) with HT have been diagnosed in the same period, 1405 female and 243 male (female-to-male ratio of 5.8:1). The proportion of children under 3 years of age was 1.15%. Two patients were younger than 1 year of age and both were boys. Four patients were between 1–2 years old, with a female-to-male ratio of 1:3. Thirteen patients were between 2–3 years old, with a female-to-male ratio of 5.5:1 (**Table 1**).

**Table 1:** Characteristics of the 19 children with HT.

Characteristic	Value
Sex	
Female	12 (63.2%)
Male	7 (36.8%)
Female/male (ratio)	1.7/1
Age (in months)	
Range	10–36
10–12	2 (10.5%)
12–24	4 (21.1%)
24–36	13 (68.4%)
Sex ratio by age (F/M)	
younger than 1 year old	0/2
1–2 years	1/3
2–3 years	5.5/1
Thyroid function status	
Overt hypothyroidism	10 (52.6%)
Subclinical hypothyroidism	7 (36.8%)
Hyperthyroidism	1 (5.3%)
Euthyroidism	1 (5.3%)
Chief complaint	
Thyroid enlargement	4 (21.1%)
Global developmental delay	4 (21.1%)
Routine screen of T1DM patients	5 (26.3%)
Growth failure	2 (10.5%)
First-degree relatives with thyroid diseases	2 (10.5%)
Poor appetite and Abdominal distension	1 (5.3%)
Accidental detection	1 (5.3%)
Positive family history	9 (47.4%)
Positive antibody	
TPOAb	17 (89.5%)
TGAb	17 (89.5%)
TPOAb + TGAb	15 (78.9%)
Other findings	
T1DM	5 (26.3%)
Turner syndrome	1 (5.3%)
Small for gestational age	1 (5.3%)
Umbilical hernia	1 (5.3%)
Autoimmune encephalitis	1 (5.3%)

### Clinical Characteristics

At the time of evaluation, one patient (5.3%) was classified as having euthyroidism, ten (52.6%) as having hypothyroidism, seven (36.8%) as having subclinical hypothyroidism, and one (5.3%) as having hyperthyroidism (**Table 1**). The average age and the female-to-male ratio was various in different subgroups, as shown in **Table 2**.

**Table 2:** Characteristics of the four subgroups at diagnosis.

	Overt hypothyroidism (n=10)	Subclinical hypothyroidism (n=7)	Hyperthyroidism (n=1)	Euthyroidism (n=1)
Female/male (ratio)	1.5/1	2.5/1	0/1	1/0
Age (in months)	23.6 ± 9.6	28.3 ± 5.6	25	36
Clinical characteristics				
Poor appetite	5	2	0	0
Goiter	5	1	0	0
Short stature	4	2	0	0
Global developmental delay	4	0	0	0
Constipation	4	0	0	0
Slow heart rate	3	0	0	0
Abdominal distension	2	0	0	0
Fatigue	2	0	0	0
Pale complexion	2	0	0	0
Routine screen of T1DM patients	1	2	1	1
First-degree relatives with thyroid disease	0	2	0	0
Biochemical parameters				
TPOAb positive	10	5	1	1
TGAb positive	9	6	1	1
TPOAb+TGAb positive	9	4	1	1
Mild anemia	4	0	0	0
AST and ALT elevated	3	1	0	0
CK elevated	3	0	0	0
Creatinine elevated	3	0	0	0
Triglyceride elevated	3	1	0	0
Cholesterol elevated	4	1	0	0
Thyroid ultrasound				
thyroid volume (ml)	5.47 ± 4.63	4.38 ± 3.57	2.23	3.59
rough echoes	9	4	1	1
uneven echoes	3	2	0	1
weak echoes	4	1	0	1
grid-like changes	0	1	0	0
nodules	2	0	0	0
Cardiac ultrasound				
Pericardial effusion	4	0	0	0

The most common reasons for doctor visits were thyroid enlargement (21.1%), global developmental delay (21.1%), and routine functional thyroid testing in patients with type 1 diabetes (T1DM; 26.3%) (**Table 1**). Initial thyroid function status stratified by reason for the initial visit are presented in **Table 3**.

**Table 3:** Initial thyroid function status stratified by reason for the initial visit in 19 children with HT.

		Goiter (n=4)	Global developmental delay(n=4)	Growth failure (n=2)	Abdominal distension (n=1)	Routine screen of T1DM patients (n=5)	First-degree relatives with thyroid diseases (n=2)	Accidental detection (n=1)
M/F	0/4		4/0	1/1	0/1	1/4	1/1	0/1
Age (in months)								
10–12	0		2	0	0	0	0	0
12–24	0		2	0	1	0	0	0
24–36	4		0	2	0	5	2	1
Thyroid function status								
Hypothyroidism	3		4	1	1	1	0	0
Subclinical hypothyroidism	1		0	1	0	2	2	1
Hyperthyroidism	0		0	0	0	1	0	0
Euthyroidism	0		0	0	0	1	0	0

Common clinical manifestations included poor appetite, goiter, short stature, global developmental delay, constipation, slow heart rate, abdominal distension, fatigue, and pale complexion. Clinical characteristics in different subgroups are presented in **Table 2**.

Other findings included Turner syndrome, small for gestational age, umbilical hernia, and autoimmune encephalitis. Nine patients (47.4%) had a familial history of a thyroid disease (**Table 1**).

Biochemical parameters and ultrasound scan result in different subgroups are presented in **Table 2**. After treatment with levothyroxine (LT4), the patients’ blood abnormalities, liver function, myocardial enzyme spectrum, renal function, and blood lipid levels all returned to normal. Additionally, pericardial effusion disappeared.

### Follow-Up and Treatment

**Figure 1:**
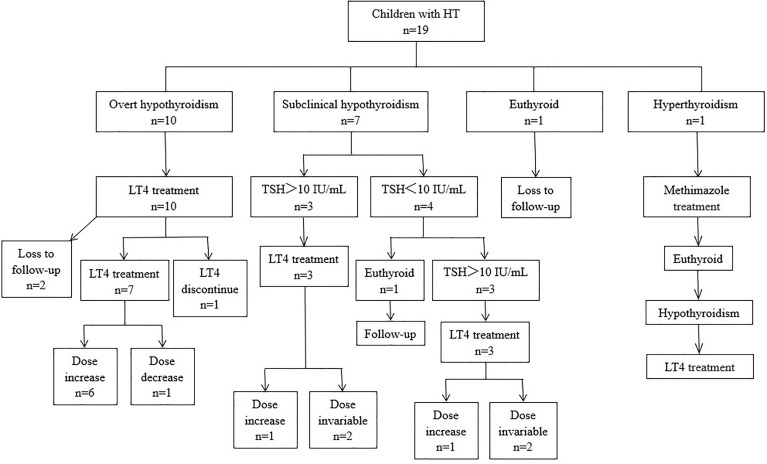
Thyroid function at presentation, treatment, and clinical courses of 19 children with HT. LT4, L-thyroxin; TSH, thyroid-stimulating hormone.

The followed-up date is shown in **Figure 1**. One patient with hypothyroidism and T1DM discontinued LT4 after 2 months of treatment and did not take the drug again. After 24 months of follow-up, the thyroid function remained normal. The only patient with hyperthyroidism was treated with methimazole at the time of initial diagnosis, and the drug dose was gradually reduced. Methimazole treatment was stopped after 11 months. Subsequently, the patient’s thyroid function remained at a normal level until the emergence of subclinical hypothyroidism, which appeared 20 months after the initial diagnosis. Treatment with LT4 was initiated 26 months after diagnosis, due to the gradual increase in TSH levels.

Four patients with global developmental delay were treated with LT4. One patient’s mental development returned to approximately the same level as that of normal age-matched children, and the remaining three patients still exhibited varying degrees of developmental delay.

Among the six patients with the clinical manifestation of short stature, four had hypothyroidism and two had subclinical hypothyroidism. All of them were treated with LT4. The recumbent length catch-up of four patients gradually increased. One patient had a shorter follow-up time (only 1 month) and there was no significant change in recumbent length. One patient with subclinical hypothyroidism had no recumbent length catch-up.

## Discussion

In this retrospective study, we examined the clinical characteristics of children under 3 years old who presented with HT and monitored the natural progression of the disease. HT rarely occurs within the first few years of life (17). Recently, an epidemiological study conducted in Spanish patients 1–16 years old (1,387 samples) revealed that the prevalence of autoimmune thyroiditis was 3.2% between the ages of 12–16 years, 1.2% between the ages of 6–12 years, and 0% between the ages of 1–6 years (2). Only a few cases in children under 3 years old have been previously reported (9–18). This study has expanded on the clinical data available on HT in children under 3 years old and provided a clinical basis for the diagnosis and treatment of HT starting at a very young age.

In a study of children and adolescents with HT, females were shown to have an increased incidence of HT with a female-to-male ratio of 4~5:1 (5, 6, 25). However, in this study, the female-to-male ratio was only 1.7:1, which was significantly lower than what has been previously reported (5, 6, 25). Further analysis of the data revealed that the male proportion was dominant in patients under 2 years old. The female-to-male ratio in 2–3-year-old patients was 5.5:1, which is similar to the previously published results (5, 6, 25). In-line with our findings, Foley et al. (9) reported four patients with HT aged between 9 months and 2 years, of which three were boys and only one was a girl. Together, these data support that boys have a higher risk of HT within the first 2 years of life.

The genetic susceptibility of HT has always been recognized. A cohort study in India revealed that compared with the general adult population, first-degree relatives of patients with HT had a 9-fold increased risk of developing HT (26). Another study showed that mothers with HT had a 32-fold increased risk of transferring autoimmune thyroiditis to their offspring (27). Therefore, in subsequent generations of mothers with HT, the thyroid function should be closely monitored even if initial screenings for congenital hypothyroidism are negative.

This study found that, at diagnosis, 52.6% of patients had overt hypothyroidism, 36.8% had subclinical hypothyroidism, 5.3% had euthyroidism, and 5.3% had hyperthyroidism. In this study, the proportion of patients with hypothyroidism was significantly higher than what has been previously reported. While the proportion of patients with subclinical hypothyroidism was relatively high, and the proportion of patients with normal thyroid function was relatively low (6, 7, 28). In previous reports of HT in infants, most patients had hypothyroidism (9–12, 14, 15, 17, 18), and only a few patients had hyperthyroidism (16), which is consistent with the results found in this study. This suggests that children below 3 years old with HT are more prone to having hypothyroidism, and clinicians should pay particular attention to early indicators.

In young children with HT, the primary reasons for doctor visits were thyroid enlargement, global developmental delay, and routine thyroid function tests in patients with T1DM. After further review of the patients’ medical history and carrying out the physical examination, it was found that many patients had anorexia and short stature as clinical manifestations. These symptoms seem to have been overlooked by the parents, leading to a delay in diagnosis and treatment. Additionally, although 21.1% of patients sought medical attention due to thyroid enlargement, and the proportion of goiter could increase to 31.6% after physical examination by physicians, this proportion was still significantly lower than that in older children with HT (25, 29). This was also one of the easily overlooked indicators of HT in children under 3 years old.

Hypothyroidism caused by HT include a variety of clinical manifestations. Global developmental delay is one of the characteristic clinical manifestations seen in infants, and it is also the clinical manifestation that causes the serious injury to the patient. In this study, global developmental delay was observed in patients 10 to 20 months old, and the age at which the children exhibited developmental delay or developmental regression was 6 months to 1 year, which is considered a critical period for the development of the nervous system. Thyroid hormone is very important for normal maturation of the central nervous system of fetuses and infants; therefore, the younger the patient is, the more likely the damage to the nervous system. Developmental abnormalities of the nervous system caused by HT-induced hypothyroidism are different from those caused by congenital hypothyroidism. Since the thyroid function of HT patients is normal in early life, they also have normal mental motor development before the onset of the disease. Evaluating the patient’s previous imaging data can help to distinguish between HT and congenital hypothyroidism. Marzuillo et al. (14) reported a patient with HT who was diagnosed at 22 months and had developmental delay. After treatment with LT4, the mental development partially recovered by the time the patient was 30 months old. As reported by Foley et al. (9), among the four patients with HT, after thyroxine treatment, one patient had the same intelligence as that of children of the same age, two patients showed only slight language development retardation, and one patient had developmental and cognitive retardation. Our results are roughly the same as those previously reported. The reasons for differences in mental motor development outcomes in children may be related to the children’s age of disease onset and the time from onset to diagnosis. The earlier the age of onset and the later the initiation of treatment, the more severe and irreversible the damage to the central nervous system.

Severe growth failure was another prominent clinical manifestation of hypothyroidism caused by HT. However, it has been reported that patients’ height can be partially improved after LT4 replacement therapy (30). In this study, six patients had severely short stature, and all received LT4 treatment. Five patients were followed-up for more than 1 year and four of them had a gradual increase in height z-scores. However, the height of one patient had not significantly improved. This patient did not have severe hypothyroidism. Therefore, it could not be ruled out that the child’s short stature was caused by other diseases, and further examination should be conducted to determine the cause.

HT is often associated with other autoimmune diseases. In this study, the most common reason for the initial doctor’s visit was that the patients with T1DM were found to have HT through a routine thyroid function examination. A recent study which included 1,053 samples indicated that there were significant differences in the types of autoimmune diseases that occur in adults and children with HT. The most common autoimmune diseases in adults were arthropathy and connective tissue diseases. However, in children and adolescents, these diseases were absent or very rare, and the most common ones were T1DM and celiac disease (28). Unfortunately, in our study, we did not test for celiac disease; this correlation should be considered in future studies and incorporated into the examinations during follow-up visits. HT is also the most common autoimmune disease in children with T1DM. It has been reported that 18.3% of children with diabetes are TPOAb positive (31). T1DM was usually rare in children under 3 years old (32). It’s worth noting that there are totally 5 patients (26.3%) associated with T1DM, were diagnosed under 3 years old, in this study.

In this study, there was one patient with Turner syndrome. Interestingly, HT is the most common autoimmune disease in patients with Turner syndrome, and its incidence gradually increases with age (23, 33). A Chinese study of 69 patients with Turner syndrome (2 months to 18 years old) indicated that HT occurred only in patients with Turner syndrome older than 5 years of age (23). Similarly, Gawlik et al. (34) reported that the age of children with Turner syndrome who were also positive for thyroid autoantibodies was above 5.5 years. This study suggests that abnormal thyroid function in children with Turner syndrome may occur at a younger age.

In order to explore the natural disease course of very young patients with HT, we performed an extended patient follow-up in this study. Presently, the results of studies on the natural course of HT in children and adolescents are inconsistent. Demirbilek et al. (35) pointed out that after an average follow-up of 50 months, 77% of patients with normal thyroid function at diagnosis were still normal, 21.1% of patients progressed to hypothyroidism, 69.5% of patients with hypothyroidism at diagnosis still had hypothyroidism, and 30.5% returned to normal thyroid function. Another study (5) showed that 40.6% of patients with subclinical hypothyroidism returned to normal function within 5 years of receiving an HT diagnosis. A recent study on the long-term evaluation of children with HT (mean 8.1 years) showed that LT4 treatment was still needed for some patients who had euthyroidism, subclinical hypothyroidism with TSH < 10 mIU/L, or subclinical hypothyroidism with TSH > 10 mIU/L, and hyperthyroidism (26%, 56%, 83-84%, and 57%, respectively), when the study was terminated (6). Several children diagnosed with hypothyroidism (16%) discontinued LT4 treatment (6). In this study, it was found that during the follow-up process, the proportion of patients who used LT4 treatment was significantly higher than that in previous studies. It is possible that the prevalence was higher due to our shorter follow-up window. On the other hand, very young patients with HT may be more likely to develop hypothyroidism, which is more difficult to recover from. As such, it is necessary to closely monitor thyroid function and once LT4 replacement therapy is initiated, medication should not be arbitrarily stopped.

This study also has certain limitations. First, the sample size was small and should be increased in future studies to help enrich the clinical data. Further, the patient follow-up period was short and more long-term assessments should be carried out to improve our understanding of the extended outcomes.

In summary, clinicians need to pay attention to the potential manifestations of HT in young children such that indicators are not overlooked and misdiagnosis can be prevented. The following aspects should be noted: (1) for patients with global developmental delay, even if thyroid function was normal in the postnatal screening, particular attention should be paid to improve thyroid examination and definitively determine whether there is hypothyroidism caused by HT; (2) for patients with T1DM, it is necessary to monitor thyroid function during the initial diagnosis and at all follow-up evaluations; and for patients with HT, blood glucose levels should be tracked and other autoimmune diseases should monitored; (3) short stature and poor appetite may be overlooked by parents of children with HT. Clinicians should perform detailed inquiries regarding medical history and careful physical examinations to avoid missed diagnosis; (4) most children with HT are in the hypothyroid or subclinical hypothyroid stage and need timely treatment; thyroid function should be closely monitored and treatment adherence should be strictly enforced for these patients; (5) after thyroxine replacement therapy, the clinical manifestations caused by hypothyroidism can be alleviated to a certain extent. Early diagnosis and treatment are essential for improving the prognosis of children.

## Data Availability Statement

The raw data supporting the conclusions of this article will be made available by the authors, without undue reservation.

## Ethics Statement

The studies involving human participants were reviewed and approved by Ethics Committee of Shengjing Hospital Affiliated with China Medical University. Written informed consent from the participants’ legal guardian/next of kin was not required to participate in this study in accordance with the national legislation and the institutional requirements.

## Author Contributions

ST collected clinical data and wrote the article. MY, DZ, and Y-jT collected clinical data. YX was responsible for the revision of the manuscript to ensure the inclusion of important intellectual content. All authors contributed to the article and approved the submitted version.

## Conflict of Interest

The authors declare that the research was conducted in the absence of any commercial or financial relationships that could be construed as a potential conflict of interest.

## Publisher’s Note

All claims expressed in this article are solely those of the authors and do not necessarily represent those of their affiliated organizations, or those of the publisher, the editors and the reviewers. Any product that may be evaluated in this article, or claim that may be made by its manufacturer, is not guaranteed or endorsed by the publisher.

## Abbreviations

HT, Hashimoto’s thyroiditis; DQ, developmental quotients; TPOAb, anti-thyroid antibodies against peroxidase; TGAb, anti-thyroid antibodies against thyroglobulin; FT4, free thyroxine; TSH, thyroid-stimulating hormone; FT3, free triiodothyronine; T1DM, type 1 diabetes; AST, aspartate aminotransferase; ALT, alanine transferase; CK, creatine kinase; CK-MB, creatine kinase MB isoenzyme; LT4, Levothyroxine.
